# Current Insights into Glutathione Depletion in Adult Septic Patients

**DOI:** 10.3390/antiox14091033

**Published:** 2025-08-22

**Authors:** Sonia Gomar, Ricardo Bou, Francisco Javier Puertas, María Miranda, Francisco Javier Romero, Belén Romero

**Affiliations:** 1 Intensive Care Unit, Hospital de Manises, 46940 Manises, Spain; gomar_son@gva.es; 2 Facultad de Medicina y Ciencias de la Salud, Universidad Católica de Valencia, 46001 Valencia, Spain; fj.puertas@ucv.es; 3 Infection Control Unit, Hospital Universitario de la Ribera, 46600 Alzira, Spain; bou_ric@gva.es; 4 Neurophysiology Unit, Hospital Universitario de la Ribera, 46600 Alzira, Spain; 5 Fundación para el Fomento de la Investigación Sanitaria y Biomédica de la Comunidad Valenciana (FISABIO), 46001 Valencia, Spain; 6 Facultad de Ciencias de la Salud, Universidad CEU Cardenal Herrera, 46115 Alfara del Patriarca, Spain; mmiranda@uch.ceu.es; 7 Hospital General de Requena, 46340 Requena, Spain; 8 MediPark AG, 4600 Olten, Switzerland

**Keywords:** sepsis, glutathione, oxidative stress, multiorgan failure

## Abstract

Sepsis is a complex condition characterized by an uncontrolled inflammatory response to infection, which can trigger multi-organ dysfunction and is associated with high mortality rates. In this context, oxidative stress plays a key role in the progression of tissue damage. Reduced glutathione (GSH), the primary non-enzymatic intracellular antioxidant, serves as a fundamental pillar in redox defense, acting as a key modulator of immune response, endothelial barrier integrity, and mitochondrial metabolism. This review explores the multifaceted role of GSH in the pathophysiology of sepsis, with emphasis on its biphasic effect on both innate and adaptive immunity, as well as its involvement in vascular alterations and mitochondrial dysfunction. The molecular mechanisms of GSH depletion during sepsis are analyzed, including excessive consumption by reactive species, disruption of its synthesis, and its intracellular compartmentalization. Additionally, the available clinical evidence in humans regarding the functional consequences of GSH loss is reviewed, particularly concerning organ failure—understood more as a bioenergetic and functional disruption than a structural one—and mortality, highlighting the methodological limitations and heterogeneity of the reported findings. Altogether, this analysis intends to provide a comprehensive view of the role of glutathione in redox dysregulation and the pathophysiological mechanisms underlying sepsis. Furthermore, it seeks to consolidate current pathophysiological and clinical knowledge to emphasize the potential role of glutathione as a prognostic marker and possible target for future therapeutic strategies in addressing this complex condition.

## 1. Introduction and Background

Sepsis is a complex and potentially life-threatening clinical syndrome, defined as organ dysfunction resulting from a dysregulated host immune response to infection [1]. This pathological process extends beyond the scope of localized infection, involving a multifactorial cascade that disrupts systemic homeostasis through profound alterations in circulatory, cellular, and metabolic systems. The inability of the host to sustain adequate tissue perfusion and effective metabolic balance progressively leads to multiple organ failure, a central phenomenon in the morbidity and mortality associated with sepsis and a recognized critical predictor of clinical outcome [1]. Its incidence and prevalence have shown a sustained increase over recent decades, driven by demographic factors such as population aging, as well as the growing complexity of medical and therapeutic interventions. Within this context, sepsis represents a major public health concern, not only due to its high mortality—reaching rates of 30–50%—but also because of its socioeconomic burden, encompassing substantial resource utilization and long-term functional and cognitive sequelae among survivors of the acute episode [2,3,4,5].

Septic shock, defined by persistent tissue hypoperfusion despite adequate fluid resuscitation, markedly increases mortality rates to alarming levels, estimated between 50% and 60% [6]. This risk escalates proportionally with the number of organs involved; thus, multiple organ dysfunction syndrome (MODS) becomes a robust prognostic indicator. Multicenter studies have documented mortality rates up to 65% when four or more organ systems are compromised, underscoring the necessity for dynamic monitoring of physiological deterioration in critically ill patients [7]. Despite advances in supportive care and therapeutic strategies, the pathophysiological mechanisms underlying sepsis remain incompletely understood and are the subject of ongoing investigation. Among the implicated processes, oxidative stress plays a central role by disrupting redox homeostasis, inducing structural cellular damage, and impairing endogenous antioxidant defenses [8,9,10,11,12].

Oxidative stress is defined as an imbalance between the production of reactive oxygen species (ROS) and the capacity of antioxidant systems to neutralize them [13]. In sepsis, massive ROS generation—driven by activated neutrophils, dysfunctional mitochondria, and enzymatic systems such as NADPH oxidase—results in direct oxidative damage to essential cellular components, including membrane lipids, structural proteins, and nucleic acids [12,14,15,16,17]. This pro-oxidant microenvironment perpetuates an exaggerated inflammatory response, disrupts endothelial function, impairs mitochondrial bioenergetics, and initiates a cascade of events culminating in MODS.

Glutathione (GSH), a tripeptide composed of glutamate, cysteine, and glycine, emerges as the primary intracellular non-enzymatic antioxidant. GSH plays a critical role in the neutralization of free radicals, maintenance of cellular redox status, and modulation of immune and inflammatory signaling pathways essential for tissue homeostasis [18,19]. However, during sepsis, GSH levels are significantly depleted due to accelerated consumption as well as impaired synthesis and recycling. This depletion exacerbates oxidative stress and directly contributes to the loss of cellular integrity, activation of apoptotic pathways, and the characteristic organ dysfunction seen in septic states.

This review focuses on the role of GSH as a key integrative axis between oxidative stress and sepsis pathophysiology: from the redox imbalance, characteristic of this critical condition, into glutathione biology and its essential physiological functions in maintaining immune homeostasis, endothelial integrity, and mitochondrial function. Furthermore, the consequences of GSH depletion are examined across multiple pathophysiological domains: from its dual influence on innate and adaptive immune responses, to its involvement in endothelial dysfunction and mitochondrial metabolic impairment—components closely linked to organ failure in sepsis. Finally, the available clinical evidence connecting GSH levels with the progression of MODS and its potential prognostic value is reviewed. This perspective supports the conceptualization of GSH not only as a marker of redox status but also as an active regulator in the progression of multiorgan damage associated with sepsis.

## 2. Oxidative Stress and Sepsis

In sepsis, multiple pathophysiological derangements converge to disrupt immunological, metabolic, and redox pathways. This acute, dysregulated host inflammatory response entails a complex crosstalk among these systems. Within this framework, oxidative stress assumes a central role: not only does it perpetuate immune activation, but it also directly mediates diffuse endothelial injury, interferes with mitochondrial function, and perturbs cellular bioenergetics, decisively contributing to the onset of multi-organ dysfunction.

During the initial phase, oxidative stress amplifies innate immunity by boosting pattern recognition receptor (PRR) signaling, notably via Toll-like receptors (TLRs) on plasma membranes and endosomal compartments of innate cells such as neutrophils, monocytes, and macrophages [20,21]. TLR activation recruits adaptors like MyD88 and TRIF, orchestrating sequential activation of IRAK, TRAF6, and the IκB kinase complex (IKK). Phosphorylation and degradation of IκB liberates NF-κB, enabling nuclear translocation and transcription of pro-inflammatory and microbicidal genes [22,23]. NF-κB upregulates cytokines (TNF-α, IL-1β, IL-6) and effector enzymes including inducible nitric oxide synthase (iNOS), cyclooxygenase-2 (COX-2), and NADPH oxidase (NOX2), driving additional ROS production. This creates a feed-forward loop: oxidative stress intensifies inflammation, risking uncontrolled immune activation and tissue damage [24,25]. Concurrently, the TRIF pathway activates interferon regulatory factors (IRFs), especially IRF3, inducing type I interferons (IFN-α/β) that modulate macrophage activation and antimicrobial gene expression—exerting both protective and pathogenic effects in sepsis [26,27].

The redox-reactive milieu also sustains inflammation via secondary release of DAMPs, including mitochondrial DNA (mtDNA), HMGB1, and extracellular ATP [28]. These DAMPs re-engage PRRs, reinforcing a positive feedback loop that escalates immune activation and tissue injury [29,30]. The severity and persistence of oxidative stress and inflammation correlate strongly with clinical severity and mortality in septic patients across multiple clinical studies, establishing them as independent prognostic biomarkers [31,32].

Beyond amplifying inflammatory signaling, oxidative stress generates direct microbicidal effectors. ROS production, principally by the NADPH oxidase-2 (NOX2) complex assembled in neutrophil and macrophage membranes, yields superoxide (O_2_^•−^), a precursor to hydrogen peroxide (H_2_O_2_) and hydroxyl radicals (^•^OH) in the presence of transition metal ions—highly cytotoxic species [33,34]. While these ROS aid pathogen clearance, they simultaneously inflict damage on host cell structures: lipid peroxidation, DNA strand breaks, and modification of structural and enzymatic proteins. Concurrently, iNOS induction in monocytes/macrophages, governed by NF-κB and other pro-inflammatory pathways, catalyzes sustained ^•^NO production from L-arginine [35]. ^•^NO reacts rapidly with O_2_^•−^ to form peroxynitrite (ONOO^−^), a potent reactive nitrogen species (RNS) that nitrates tyrosine residues, inactivates mitochondrial enzymes such as aconitase, and promotes lipid peroxidation—compromising both structural integrity and bioenergetic function [36,37].

Adaptive immunity is also profoundly impaired. T- and B-lymphocytes undergo ROS/RNS-mediated apoptosis via both intrinsic (mitochondrial damage, cytochrome c release, caspase-9/3 activation) and extrinsic (Fas/FasL-mediated caspase-8 activation) pathways [38,39]. Pronounced lymphopenia—especially CD4^+^ T cells—weakens adaptive responsiveness. Surviving T cells adopt an exhausted phenotype, with overexpression of inhibitory receptors (PD-1, CTLA-4, LAG-3), limiting clonal expansion, effector cytokine production (IFN-γ, IL-2), and memory cell development [40]. ROS-exposed antigen-presenting cells upregulate PD-L1, amplifying inhibitory signaling and promoting immunosuppression [41,42]. Hypoxia and oxidative stress also induce metabolic reprogramming in T cells towards inefficient aerobic glycolysis, undermining oxidative phosphorylation and mitochondrial biogenesis required for memory T cell function [43,44]. Inhibition of regulatory pathways such as AMPK/SIRT1 [45] and intracellular antioxidant depletion further magnify redox damage and immune dysfunction [46,47].

In B cells, oxidative stress damages DNA and activates the p53–ATM/Chk2 axis, causing cell-cycle arrest and apoptosis, thus reducing clonal quantity and diversity [48,49,50]. Germinal center reactions are impaired: ROS inhibit activation-induced cytidine deaminase (AID), restricting somatic hypermutation and class switching, and thereby reducing antibody specificity and affinity [51]. ROS also perturb BCR signaling and downstream PI3K/Akt and MAPK pathways—altering kinase and phosphatase activity and adapter proteins—leading to impaired proliferation, survival, and antibody secretion [48]. At the epigenetic level, oxidative stress suppresses adaptive immune gene expression by inhibiting histone deacetylases (HDACs) and demethylases like TET2 and DNMT3A [52,53]. These modifications limit functional plasticity of lymphocytes, leading to an immunological anergy and tolerance status.

These adaptive dysfunction mechanisms occur within the compensatory anti-inflammatory response syndrome (CARS): active immunosuppression marked by lymphocyte depletion, sustained inhibitory receptor expression, tolerogenic antigen-presenting cell phenotypes, and profound intracellular signaling and epigenetic reprogramming mediated by oxidative stress [40,54,55,56]. Prolongation of CARS impairs immune surveillance, predisposes the body to opportunistic infections and contributes to the PICS phenotype—persistent inflammation, immunosuppression, and catabolism—closely associated with poor outcomes [57,58].

Meanwhile, oxidative stress exerts a critical impact on the endothelium, revealing another dimension of sepsis pathophysiology: vascular dysfunction. Normally, vascular endothelium regulates vasomotor tone, hemostasis, permeability, and immune response. Under septic conditions, it becomes both a generator and target of ROS. Activation by PAMPs and DAMPs induces endothelial ROS from dysfunctional mitochondria, uncoupled endothelial NOS (eNOS), and NADPH oxidase—particularly NOX4, whose upregulation by LPS increases vascular permeability—alongside contributions from xanthine oxidase and lipoxygenases [16]. A major consequence is reduced nitric oxide bioavailability, impairing vasodilation, anti-platelet effects, and anti-inflammatory signaling [35]. While this eNOS-related ^•^NO depletion may dominate in early sepsis, septic shock is generally driven by robust inducible NOS (iNOS) upregulation, causing excessive ^•^NO production, systemic vasodilation, and hypotension [35]. Interaction between O_2_^•−^ and ^•^NO forms ONOO^−^, which inactivates ^•^NO, nitrates proteins, inactivates antioxidant enzymes (SOD, catalase, GPX4), and promotes lipid peroxidation, as mentioned above [36,37].

This cascade disrupts endothelial barrier integrity: oxidative stress perturbs tight and *adherens* junctions, redistributing ZO-1, occludin, and VE-cadherin to increase permeability and facilitate immune cell extravasation [16,59,60]. Glycocalyx degradation—mediated by ROS, heparanases, metalloproteinases, and inflammatory cytokines—exposes the underlying endothelium, enhancing leukocyte adhesion and vascular injury, while impairing its barrier, signaling, and anti-inflammatory functions [16,61,62]. NF-κB-driven expression of adhesion molecules (ICAM-1, VCAM-1, E-selectin) further promotes leukocyte–endothelial interactions and transmigration [16,59,60]. Additionally, endothelial apoptosis due to oxidative damage to membranes and mitochondrial proteins exacerbates microvascular dysfunction and microhemorrhage [63,64]. Endothelium shifts to a procoagulant phenotype, with increased tissue factor, reduced anticoagulants (thrombomodulin, activated protein C), platelet activation, microthrombi formation, and impaired capillary perfusion. Inhibited fibrinolysis—via upregulated PAI-1—perpetuates thrombosis [65]. This vascular injury intensifies microcirculatory dysfunction and accelerates multiorgan damage [59,66,67]. Yet organ dysfunction in sepsis is not solely due to hypoperfusion, but also to impaired oxygen utilization at the cellular level—a phenomenon termed cytopathic hypoxia [68].

Mitochondria thus play a central role, not just as energy generators via oxidative phosphorylation, but also as both major ROS sources and critical targets. Mitochondrial dysfunction may occur even when tissue oxygenation appears adequate, perpetuating cellular damage, apoptotic activation, and organ dysfunction. Clinical studies correlate increased oxidative stress, mitochondrial bioenergetic disruption, and sepsis severity [32,69,70,71]. Morphological autopsy findings include mitochondrial swelling, loss of cristae, matrix depletion, intramitochondrial vesiculation, and membrane rupture [72] caused by excessive ROS/RNS, respiratory chain dysfunction, and intracellular calcium overload [73]. Functionally, sepsis impairs electron transport—especially complexes I and III—causing electron leakage and enhanced superoxide production. ROS damage lipids, proteins, and mtDNA, compromising mitochondrial protein synthesis and perpetuating energy deficits. Released mtDNA, structurally similar to bacterial DNA, can act as a DAMP—engaging TLR9 in endosomes and activating the NLRP3 inflammasome and the cGAS–STING DNA sensing pathway—inducing IL-1β, IL-18, and type I interferons, amplifying systemic inflammation and organ dysfunction [74,75,76,77,78].

**Figure 1:**
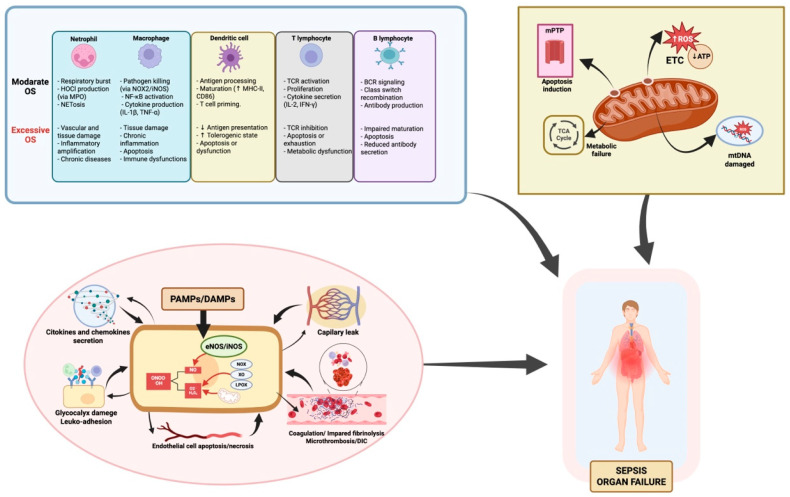
**Oxidative stress in sepsis: immune dysfunction, endothelial injury, and mitochondrial damage as drivers of functional organ failure.** This figure summarizes how oxidative stress (OS) contributes to organ failure in sepsis through three main mechanisms: **1. Immune dysregulation.** OS alters both innate and adaptive immune responses. While moderate ROS support pathogen clearance, antigen presentation, and cytokine release, excess ROS disrupt homeostasis, causing immune cell exhaustion, apoptosis, and dysfunctional signaling. The degree of immune impairment varies depending on the cell type and ROS load, affecting macrophages, neutrophils, T and B lymphocytes, and dendritic cells differently. **2. Endothelial injury.** Excessive ROS disrupt the endothelial barrier, increasing vascular permeability and edema. ROS inhibit eNOS activity, reducing NO availability critical for vasodilation and vascular homeostasis. OS-induced endothelial activation upregulates adhesion molecules, promoting leukocyte adhesion and transmigration, exacerbating inflammation. This damage contributes to microvascular thrombosis, DIC, and impaired tissue perfusion, further driving organ dysfunction. **3. Mitochondrial Damage**. Mitochondria are both a source and a target of ROS in sepsis. OS disrupts the mitochondrial respiratory chain, reducing ATP production and increasing mitochondrial ROS, creating a self-perpetuating cycle. mtDNA damage and loss of membrane potential, including mPTP opening, trigger the release of pro-apoptotic factors, leading to cell death. This mitochondrial dysfunction causes energy failure and metabolic impairment, key drivers of organ dysfunction in sepsis. Additionally, impaired mitophagy and biogenesis worsen mitochondrial damage, delaying recovery. **Abbreviations:** ATP: adenosine triphosphate; BCR: B cell receptor; CD86: cluster of differentiation 86; DAMPs: damage-associated molecular patterns; DIC: disseminated intravascular coagulation; eNOS: endothelial nitric oxide synthase; ETC: electron transport chain; H_2_O_2_: hydrogen peroxide; HOCl: hypochlorous acid; iNOS: inducible nitric oxide synthase; LOX: lipoxygenase; MHC-II: major histocompatibility complex class II; MPO: myeloperoxidase; mtDNA: mitochondrial DNA; mPTP: mitochondrial permeability transition pore; NADPH oxidase (NOX); NOX2: NADPH oxidase 2; NETosis: neutrophil extracellular trap formation; NF-κB: nuclear factor kappa-light-chain-enhancer of activated B cells; NO: nitric oxide; O_2_^•−^: superoxide anion; OH·: hydroxyl radical; ONOO^−^: peroxynitrite; OS: oxidative stress; PAMPs: pathogen-associated molecular patterns; ROS: reactive oxygen species; TCA cycle: tricarboxylic acid cycle; TCR: T cell receptor; XO: xanthine oxidase. Element related to endothelial dysfunction adapted from Ref. [16]; created in BioRender. Gomar, S. (2025) https://BioRender.com/m92v841, accessed on 20 August 2025.

The mitochondrial permeability transition pore (mPTP) may open, disrupting membrane potential and releasing cytochrome c, triggering intrinsic apoptosis [79,80,81]. Excessive mitochondrial fragmentation and impaired mitophagy—due to disrupted fission/fusion dynamics—hinder clearance of damaged mitochondria, further compromising cellular function [82,83,84,85,86]. To compensate for impaired mitochondrial efficiency, cells often undergo metabolic reprogramming, shifting from oxidative phosphorylation to accelerated aerobic glycolysis [87,88]. While this supports minimal function temporarily, it cannot meet the energy demands of ATP-dependent organs, contributing to organ compromise. Histopathological findings show surprisingly limited structural cell death despite severe clinical deterioration [89]. Recovery of mitochondrial respiratory function correlates with improved organ outcome in survivors [32,90], suggesting that organ dysfunction is largely functional—a reversible “cellular hibernation” in response to bioenergetic stress. However, if prolonged, this state may transition to irreversible organ failure and poorer prognosis (Figure 1).

In this context, redox balance is not solely defined by the accumulation of reactive oxygen and nitrogen species (ROS/RNS), but rather by the cellular antioxidant capacity to neutralize them and repair the resulting damage. Cells are equipped with sophisticated antioxidant defense systems, including key enzymes such as superoxide dismutase (SOD), which catalyzes the dismutation of superoxide anion (O_2_^•−^) into hydrogen peroxide (H_2_O_2_); catalase (CAT), which converts H_2_O_2_ into water and molecular oxygen; and glutathione peroxidase (GPx), which detoxifies hydroperoxides using reduced glutathione (GSH) as a critical cofactor.

However, the overwhelming inflammatory response, coupled with endothelial and mitochondrial dysfunction, and dysregulated transcriptional responses mediated by redox-sensitive factors, leads to a progressive deterioration of these antioxidant defenses. Notably, inhibition of the Keap1–Nrf2 pathway limits the transcriptional induction of antioxidant enzymes, while GSH depletion and reduced enzymatic activity impair the cellular capacity to detoxify peroxides and repair oxidative damage [12,91,92,93]. These cumulative alterations converge into a collapse of the antioxidant system, exacerbating tissue injury, perpetuating inflammation, and accelerating the progression toward multiple organ dysfunction.

Measurement of reduced glutathione (GSH) provides a more accurate assessment of the cellular antioxidant status than enzymatic activity alone, which may not fully reflect the dynamic nature of oxidative stress. Accordingly, herein we focus on the critical role of GSH in sepsis, and explore the pathophysiological consequences derived from its depletion.

## 3. Biology and Function of Glutathione

### 3.1. Intracellular Dynamics and Functional Roles of Glutathione

Glutathione (γ-L-glutamyl-L-cysteinyl-glycine), composed of glutamic acid, cysteine, and glycine, is the most abundant water-soluble tripeptide in cells and serves as the principal low-molecular-weight antioxidant synthesized endogenously. Its synthesis, transport, and catabolism occur via a series of enzymatic steps and membrane transport processes collectively referred to as the γ-glutamyl cycle, which requires the consumption of three ATP molecules. *De novo* biosynthesis of GSH occurs in the cytosol of virtually all cells via a two-step, ATP-dependent enzymatic pathway [94]. In the first step, glutamate and cysteine are enzymatically combined by glutamate-cysteine ligase (GCL)—also known as γ-glutamylcysteine synthetase—to form the γ-glutamylcysteine dipeptide. This reaction creates an amide linkage between the γ-carboxyl group of glutamate and the amino group of cysteine, distinguishing it from conventional peptide bonds [95]. GCL is a heterodimeric enzyme composed of a catalytic subunit (GCLC, approximately 73 kDa) and a modulatory subunit (GCLM, approximately 30 kDa). While GCLC provides enzymatic activity, GCLM enhances substrate affinity, catalytic efficiency, and enzyme stability. GCL activity is subject to feedback inhibition: intracellular GSH competes with glutamate for the active site of GCLC, providing a homeostatic control mechanism [96]. In the second step, γ-glutamylcysteine is conjugated with glycine by glutathione synthetase (GS), a homodimer (approximately 52 kDa per subunit), forming the reduced tripeptide GSH. Unlike GCL, GS activity is not regulated by intracellular GSH levels and thus does not constitute a physiological control point in GSH biosynthesis. The normal synthesis rate of GSH is determined primarily by: (a) GCL activity, recognized as the rate-limiting step [97], and (b) intracellular cysteine availability, the scarcest precursor under physiological conditions, making it the chief regulator of GSH synthetic flux [98,99].

Although synthesis occurs in the cytosol only, a significant fraction of intracellular GSH is rapidly exported to the extracellular space via ATP-dependent transporters [100]. There, γ-glutamyl transpeptidase (GGT) catalyzes its degradation into γ-glutamyl and cysteinyl-glycine fractions [101]. The γ-glutamyl moiety transfers to an acceptor amino acid to form a γ-glutamyl-amino acid, which can be internalized by recipient cells. Inside, it is metabolized to release the amino acid and 5-oxoproline, converted into glutamate and reused for GSH biosynthesis [18]. Meanwhile, cysteinyl-glycine is cleaved by a dipeptidase to cysteine and glycine; the resulting cysteine is recycled for new GSH synthesis.

This γ-glutamyl cycle enables continuous cysteine supply. Intracellular GSH half-life is short—approximately 2 to 6 h. In erythrocytes, turnover ranges from 50% to 100% per day, whereas in the liver (the primary storage and synthesis organ), rates may exceed 300–400% [102]. Concurrently, GSH is distributed to subcellular compartments acting as its reservoirs. Approximately 80–85% resides in the cytosol, 10–15% in mitochondria, and smaller amounts in the nucleus and endoplasmic reticulum [103]. In these compartments, GSH exists either free or covalently protein-bound. Free, reduced GSH—functionally active—comprises roughly 90% of total glutathione, reaching intracellular concentrations between 1 and 10 mM [104,105,106]. The remainder is oxidized glutathione (GSSG), formed by the disulfide linkage of two GSH molecules following electron donation from the thiol group to ROS, a reaction mediated by glutathione peroxidase (GPx) [107]. GSSG is recycled to GSH via glutathione reductase (GR), using NADPH—primarily generated via the pentose phosphate pathway—as a cofactor [108]. The GSH/GSSG ratio is a key indicator of redox status, with decreases indicating oxidative stress [19].

Beyond GR-mediated recycling, GSSG may also be degraded extracellularly via GGT, forming mono-(desGlu)-GSSG, which is further processed into CySS-bis-Gly and CySSG by GGT and dipeptidase activity, respectively. These breakdown products yield γ-glutamyl residues and glycine, enhancing amino acid recapture and reutilization in *de novo* GSH synthesis [106,109]. Notably, in the endoplasmic reticulum, glutathione predominantly exists as GSSG, contributing to the oxidizing environment necessary for proper protein folding and secretory assembly [110].

Among subcellular pools, mitochondria serve as a critical GSH reservoir, containing 10–15% of total intracellular GSH at concentrations up to ~12 mM—higher than in the cytosol [111]. This is vital because mitochondria lack catalase, making GSH the principal defense against hydrogen peroxide generated during oxidative processes in the matrix [112]. Since the mitochondrion cannot synthesize GSH, all mitochondrial GSH derives from cytosolic transport via specific anion exchangers in the inner mitochondrial membrane, namely the dicarboxylate and 2-oxoglutarate carriers [113,114,115,116]. This import ensures effective mitochondrial redox balance and is indispensable for cell survival [18,117]. Systemically, the liver is the principal site of GSH synthesis and storage. It uniquely facilitates cysteine production from methionine via the transsulfuration pathway [102,118]. Hepatic GSH is the main source of plasma GSH, which exists at concentrations between 10 and 30 µM [119]. Plasma GSH has an extremely short half-life (1–3 min) due to rapid extracellular catabolism and interorgan reutilization [19,120].

Functional roles of GSH include the following:

- Maintaining intracellular redox homeostasis as the primary non-enzymatic antioxidant, directly neutralizing ROS/RNS and preserving cellular integrity via GPx-mediated peroxide reduction and GR-mediated recycling [18].
- Regulating metabolic processes, amino acid transport, signal transduction, gene expression, mitochondrial integrity, and apoptosis prevention by inhibiting cytochrome c release and caspase activation.
- Serving as a cysteine reservoir and transporter via the γ-glutamyl cycle, protecting thiol groups from extracellular oxidation.
- Detoxifying xenobiotics through conjugation (via glutathione-S-transferase), followed by further processing and excretion as mercapturic acid derivatives [117].

### 3.2. Mechanisms of Intracellular Glutathione Depletion in Sepsis

During sepsis, systemic, sustained oxidative stress triggers critical intracellular depletion of reduced glutathione (GSH) and alters its subcellular distribution [121], compromising antioxidant defense capacity. Key mechanisms include the following:

- Enhanced consumption of GSH via detoxification of peroxides is mediated by GPx, increasing GSSG levels. The regenerative system involving GR and NADPH is impaired by metabolic dysfunction, direct oxidative inhibition of GR, and NADPH depletion, resulting in GSH/GSSG imbalance and redox collapse.
- Increased GSH export via ATP-binding transporters (e.g., MRPs) supports precursor reuse and eliminates toxic xenobiotic conjugates; however, this accelerates intracellular GSH loss. In the extracellular milieu, GSH may become inactivated by reacting with RNS to form S-nitrosoglutathione (GSNO) or by protein glutathionylation (PSSG), further depleting functional GSH.

*De novo* synthesis is compromised at multiple levels:

- GCL (the rate-limiting enzyme) may be genetically or epigenetically impaired under sepsis, exacerbated by inflammatory mediators and metabolic stress. Caspase-mediated degradation of GCL during apoptosis further reduces synthetic capacity.
- Cysteine availability declines due to accelerated catabolism, reduced absorption, and hepatic dysfunction, limiting GSH biosynthetic flux.

GCL gene expression is highly regulated: ROS, nitric oxide, IL-1, TNF-α, thyroid hormone, growth hormone, vitamin D, progesterone, and low GSH/GSSG ratio induce transcription, whereas hyperglycemia, glucocorticoids, glucagon, catecholamines, and erythropoietin suppress it [122,123,124,125], further limiting replenishment.

Subcellular compartmentalization of GSH is also disrupted:

- Mitochondrial GSH import is hindered by ROS-mediated damage to transporters, impairing mitochondrial redox protection.
- Nuclear GSH diffusion may be altered by oxidative overload.
- Endoplasmic reticulum GSH transport remains incompletely characterized but likely vulnerable to oxidative conditions.

Clinical factors such as malnutrition, prolonged immobilization, obesity, and insulin resistance exacerbate GSH metabolism dysregulation, promoting ROS production, reducing hepatic GSH, and amplifying endothelial and inflammatory dysfunction [126,127]. In early sepsis, cytokines like IL-1 and TNF-α upregulate GPx activity, increasing GSH consumption. While a short-term compensatory synthesis increase may occur, sustained inflammation leads to persistent GSH depletion, reinforcing oxidative and inflammatory stress [128,129].

**Figure 2:**
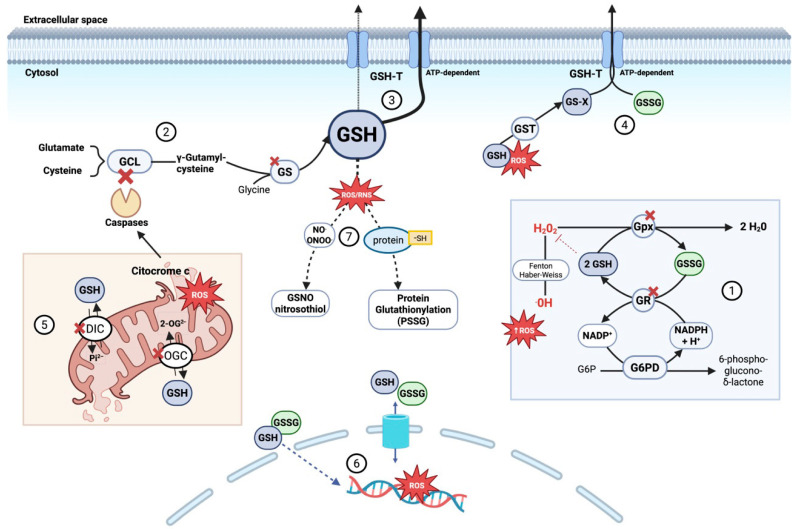
**Mechanisms of intracellular glutathione (GSH) depletion and compartmentalization disruption during sepsis.** During sepsis, GSH depletion contributes to redox imbalance and cell death through several converging mechanisms: **(1)** Increased oxidative stress accelerates GSH consumption by glutathione peroxidases detoxifying peroxides, forming GSSG. Although glutathione reductase recycles GSSG to GSH in an NADPH-dependent process, sustained stress overwhelms this system. **(2)** *De novo* GSH synthesis is impaired due to GLC degradation triggered by mitochondrial cytochrome c release and caspase activation, combined with limited availability of precursor amino acids (glutamate, cysteine, glycine). Glutathione synthetase activity may also be reduced due to oxidative modifications and altered expression during sepsis. **(3)** Enhanced ATP-dependent export of GSH via glutathione transporters (GSH-T) reduces intracellular GSH, disrupting redox balance. **(4)** Accumulated glutathione conjugates (GS-X)—formed by GSH binding to toxins and ROS via glutathione S-transferase (GST)—along with increased oxidized glutathione (GSSG) overwhelm ATP-dependent GSH-T, impairing export and causing intracellular accumulation that enhances oxidative damage. **(5)** Impaired mitochondrial glutathione compartmentalization, due to dysfunction of key carriers such as DIC and OGC, limits GSH import into the matrix. This results from oxidative damage, membrane depolarization, and inhibitory post-translational modifications. Consequently, mitochondria become dysfunctional, both generating ROS and becoming more susceptible to oxidative stress, which triggers permeability transition and disrupts ATP synthesis. **(6)** In sepsis, cytosolic redox imbalance promotes glutathione transport into the nucleus, reducing nuclear GSH and increasing GSSG levels, thereby enhancing oxidative damage to nuclear DNA. **(7)** Elevated reactive nitrogen species promote the formation of S-nitrosoglutathione (GSNO, a reservoir and scavenger of nitric oxide and peroxynitrite). In parallel, protein S-glutathionylation—where glutathione covalently binds to protein thiol groups (protein-SH)—protects proteins from irreversible oxidation and modulates their function. Both processes consume GSH, limiting its availability for antioxidant defense and aggravating oxidative stress in sepsis. **Abbreviations:** 2-OG^2−^ (2-oxoglutarate anión), DIC (dicarboxylate carrier), G6PD (glucose-6-phosphate dehydrogenase), g6P (glucose-6-phosphate), GCL (glutamate-cysteine ligase), GS (glutathione synthetase), GSH (glutathione reduced form), GSH-T (ATP-dependent glutathione transporter), GST (glutathione S-transferase), GSSG (glutathione oxidized form), GS-X (glutathione conjugates), GSNO (S-nitrosoglutathione), GR (glutathione reductase), H^+^ (proton), H_2_O_2_ (hydrogen peroxide), NADP^+^ (nicotinamide adenine dinucleotide phosphate oxidized form), NADPH (nicotinamide adenine dinucleotide phosphate reduced form), NO (nitric oxide), OGC (2-oxoglutarate carrier), ^•^OH (hydroxyl radical), Pi^2−^ (inorganic phosphate anión), PSSG (protein S-glutathionylation) and ROS (reactive oxygen species). Adapted with permission from Ref. [121]. Copyright 2012 Mary Ann Liebert, Inc. Created in BioRender. Gomar, S. (2025) https://BioRender.com/m92v841, accessed on 20 August 2025.

Altogether, intracellular GSH depletion during sepsis arises from a complex interplay of increased consumption, impaired synthesis and recycling, disrupted distribution, and enhanced export or inactivation. This loss not only weakens cellular antioxidant defenses but also compromises apoptosis regulation, inflammatory signaling modulation, and xenobiotic detoxification. Consequently, disruption of glutathione homeostasis acts as an amplifier of tissue injury and progression toward organ failure in septic patients (Figure 2).

## 4. Consequences of Glutathione Depletion in Sepsis

### 4.1. Glutathione as a Key Redox Immunological Modulator in Sepsis: Biphasic Impact on Innate and Adaptive Immune Responses

Glutathione (GSH), the principal intracellular thiol, acts as an essential redox modulator in both innate and adaptive immune responses. Its role extends beyond neutralizing reactive oxygen species (ROS), actively participating in immune signal transduction, gene expression, cellular differentiation, and cytokine production [130,131]. The reduction in intracellular GSH levels favors persistent activation of nuclear factor kappa B (NF-κB), resulting in the overproduction of proinflammatory cytokines such as TNF-α, IL-1β, and IL-6 [132,133]. This redox imbalance also enhances NLRP3 inflammasome activation, promoting massive and uncontrolled secretion of IL-1β and IL-18, central components of the so-called “cytokine storm” [134].

Experimental models have correlated this GSH deficiency-dependent inflammatory cascade with excessive and disorganized neutrophil (PMN) infiltration in pulmonary parenchyma, accompanied by insufficient migration of these cells toward the primary infection site, contributing to dysfunctional immune responses [135]. Concurrently, GSH depletion interferes with macrophage functionality, impairing their polarization toward the M1 phenotype, characterized by microbicidal activity and production of ROS essential for pathogen clearance. This disruption significantly reduces phagocytic capacity and efficient antigen presentation, promoting a shift toward M2-like phenotypes, associated with an immunosuppressive state that limits tissue damage but diminishes the host’s antimicrobial capacity [132,136,137].

At the molecular level, GSH directly participates in antigen processing by reducing disulfide bonds in endocytosed proteins within endosomes. This step is essential for proper protein denaturation and subsequent generation of immunodominant epitopes, which are presented on major histocompatibility complex class II (MHC-II) molecules to activate CD4^+^ T lymphocytes [138,139,140]. Under GSH depletion conditions, antigen-presenting cells (APCs) such as macrophages and dendritic cells show reduced MHC-II expression and decreased IL-12 production, a cytokine crucial for Th1 polarization of CD4^+^ T cells. This deficiency promotes a shift toward a tolerogenic Th2 profile, characterized by increased IL-4 and IL-10 production, contributing to immunosuppression and negative modulation of the inflammatory response [141,142].

In the context of adaptive immunity, GSH availability is critical for T lymphocyte clonal proliferation by maintaining cellular redox homeostasis and preventing activation of oxidative stress-dependent apoptotic pathways [131,143,144]. GSH synthesis critically depends on cysteine supply, a limiting amino acid whose uptake is mediated by macrophages and lymphoid lineage cells [131,145]. In septic patients, progressive GSH loss has been documented in CD4^+^ and CD8^+^ lymphocytes, correlating with increased apoptosis and sustained reduction in the number and function of these cells, contributing to secondary immunosuppression [41,54,89,146]. Various studies have shown that reduced GSH levels are associated with decreased interleukin-2 (IL-2) synthesis, a key cytokine for T lymphocyte clonal expansion, and with the inability to sustain an effective immune response against pathogens [147,148]. Additionally, active extrusion of GSH into the extracellular milieu under immune stress acts as an additional immunosuppressive mechanism independent of ROS production, hindering immune reconstitution [121,149].

Finally, neutrophils, essential for the early innate immune response, suffer notable functional impairments during GSH deficiency states. A decrease in chemotaxis, oxidative burst capacity, and overall microbicidal activity is observed, facilitating bacterial persistence and amplifying tissue inflammatory damage [150,151,152,153].

Altogether, available experimental and clinical evidence positions GSH as an essential component of immunoregulation during sepsis. Its depletion acts as a trigger and perpetuator of both dysregulated inflammation in early phases and profound immunosuppression characteristic of late-stage syndrome. This dual pathogenic role confers GSH a central function in the immune pathophysiology of sepsis, justifying its consideration as a potential therapeutic target.

### 4.2. Glutathione, Endothelium, and Mitochondria: Intertwined Mechanisms in the Progression of Septic Organ Dysfunction

In the complex pathophysiology of sepsis, glutathione (GSH) deficiency emerges as a central element triggering a cascade of structural and functional alterations affecting multiple cellular and tissue levels. The convergence of endothelial damage, tissue hypoperfusion, and mitochondrial dysfunction creates a state of dysoxia that precipitates the multiorgan failure characteristic of sepsis.

Endothelial dysfunction in sepsis is not merely an epiphenomenon of systemic inflammation but a primary pathological event that promotes an inflammatory, procoagulant, and locally vasoconstrictive state contributing to the progression of multiorgan failure [59,154,155]. One of the key mechanisms involved is the post-translational modification of endothelial nitric oxide synthase (eNOS) via S-glutathionylation, triggered by an increased GSSG/GSH ratio. This modification leads to eNOS uncoupling, significantly reducing nitric oxide (^•^NO) production and concomitantly increasing superoxide radical generation [156]. The consequent decrease in ^•^NO bioavailability not only impairs vasodilation but also promotes platelet aggregation, leukocyte adhesion, and the expression of adhesion molecules such as ICAM-1 and VCAM-1, amplifying vascular dysfunction [35].

Simultaneously, loss of function of glutathione peroxidase 1 (GPx-1), an intracellular enzyme that detoxifies H_2_O_2_ and lipid peroxides using GSH as a cofactor, favors a pro-oxidant redox environment, increasing ROS and peroxynitrite production while reducing ^•^NO bioavailability. In GPx-1 knockout murine models, the vasodilatory response to acetylcholine and bradykinin is reversed, resulting in paradoxical local vasoconstriction [157]. Elevated isoprostanes (iPF2α-III) and nitrotyrosines in the aorta and plasma confirm systemic oxidative stress [158]. Interventions that restore intracellular GSH levels, such as administration of L-2-oxothiazolidine-4-carboxylate (OTC), have demonstrated recovery of vascular tone, establishing a direct causal link between GSH deficiency and endothelial dysfunction [157]. While these mechanisms dominate at the microvascular level in early or localized sepsis, progression to septic shock is characterized by robust inducible NOS (iNOS) induction, leading to massive ^•^NO release, systemic vasorelaxation, hypotension, and diminished responsiveness to vasoactive stimuli, bridging the gap between experimental observations and the clinical hemodynamic collapse observed in patients [35,157].

In cultures of human microvascular endothelial cells (HMVECs), GPx-1 absence intensifies the proinflammatory response induced by TNF-α. This manifests as sustained activation of MAPK pathways (p38, ERK, JNK), IκB degradation, NF-κB activation, and increased expression of adhesion molecules VCAM-1 and ICAM-1, contributing to loss of endothelial barrier integrity [159]. Likewise, GSH deficiency interferes with key signaling pathways such as PI3K/Akt/eNOS, reducing eNOS phosphorylation and ^•^NO production, critical for preserving vascular function, permeability, tone, and processes like angiogenesis and vascular remodeling [160,161,162].

These mechanisms converge into profound microvascular dysfunction that not only perpetuates systemic inflammation and oxidative stress but also generates maldistributed tissue perfusion characterized by simultaneous areas of ischemia, capillary stasis, and functional shunts. This altered vascular dynamics impose limitations on effective cellular oxygen and nutrient delivery. However, beyond oxygen deficiency, cytopathic hypoxia emerges as the core of mitochondrial bioenergetic failure, characterized by an inability to utilize available oxygen, resulting in significant ATP production reduction and compromised cellular function without overt structural damage.

The mitochondrion, besides being the main source of cellular energy through coupling of the electron transport chain (ETC) and oxidative phosphorylation (OXPHOS), is also the largest intracellular ROS generator, particularly at complexes I and III [163,164]. Superoxide anion (O_2_^•−^), the primary ROS, is exponentially increased during sepsis due to disrupted electron flow, mitochondrial membrane potential (Δψm) collapse, and generalized cellular hypoxia. This superoxide is rapidly dismutated to hydrogen peroxide (H_2_O_2_), whose neutralization in the mitochondrial matrix depends mainly on two NADPH-dependent antioxidant systems: the glutathione system and the thioredoxin system. Lowes et al. evaluated the relative contribution of both systems in endothelial cells exposed to lipopolysaccharide (LPS) and peptidoglycan (PepG) [165]. Specific inhibition of mitochondrial thioredoxin 2 (TRX-2) caused marked ATP production decrease, increased anaerobic metabolism (lactate elevation), oxygen consumption reduction, and caspase 3/7 activation, reflecting more severe mitochondrial dysfunction than observed with mitochondrial glutathione system (mGSH) inhibition. These findings suggest that under intense oxidative stress, TRX-2 is more vulnerable to inactivation, while the glutathione system maintains relatively superior antioxidant capacity and mitochondrial functionality.

Mitochondrial glutathione (mGSH) is therefore a critical regulator of mitochondrial redox balance and organelle-specific function. Since it is not synthesized *de novo* within mitochondria, its concentration depends entirely on import from the cytosol, mediated by specific inner mitochondrial membrane transporters such as the oxoglutarate carrier (OGC) and dicarboxylate carrier (DIC), which facilitate GSH exchange for anions like 2-oxoglutarate or phosphate [166,167,168]. During sepsis, alterations occur in the inner mitochondrial membrane lipid composition, notably increased cholesterol content. This accumulation modifies membrane fluidity and dynamics, negatively affecting mGSH transporter function and reducing glutathione import capacity into the mitochondrial matrix. This process has been documented in studies like that of Ha et al., who showed that cholesterol overload in macrophage mitochondria induces mitochondrial hyperpolarization and increased ROS production [169].

The importance of mitochondrial glutathione (mGSH) is evident in its direct antioxidant role, detoxifying hydrogen peroxide and maintaining the integrity of mitochondrial lipids, proteins, and mitochondrial DNA (mtDNA) [170]. Glutathione peroxidase 1 (GPx1), the most abundant mitochondrial isoform, catalyzes the reduction of H_2_O_2_ to water, utilizing GSH and generating glutathione disulfide (GSSG), which must be recycled back to GSH by the action of glutathione reductase (GR) with NADPH as the electron donor [171,172]. In sepsis, the decrease in NADPH along with the accumulation of GSSG limits GSH regeneration, which, combined with reduced glutathione reductase (GR) activity, allows H_2_O_2_ to persist and generate hydroxyl radicals (^•^OH), promoting lipid peroxidation and damage to proteins and mtDNA [173,174]. Moreover, GSSG cannot be easily exported out of the mitochondria [175,176]. This accumulation favors protein glutathionylation, a reversible post-translational modification in which a glutathione residue (GSH) covalently binds to cysteine thiol groups. This modification transiently protects critical thiol groups from irreversible oxidation, but its persistence or excess can alter the structural and catalytic function of proteins essential for cellular bioenergetics.

The glutaredoxin (Grx) protein family, especially Grx2 located in the mitochondria, dynamically regulates this process by catalyzing both glutathionylation and deglutathionylation of glutathione groups on proteins, depending on the local redox state reflected by the GSH/GSSG ratio [177,178]. Under physiological conditions, Grx2 maintains mitochondrial functionality by deglutathionylating key proteins such as respiratory chain complex I, UCP3 (uncoupling protein 3), and 2-oxoglutarate dehydrogenase (OGDH) [177,178]. However, under intense oxidative stress, the collapse of mitochondrial redox balance can reduce Grx2 activity or alter its catalytic specificity, thereby favoring the accumulation of glutathione-conjugated proteins [179]. This accumulation compromises the functionality of key metabolic pathways, exacerbating mitochondrial dysfunction in tissues with high energy demands, which is directly associated with the progression of tissue injury in acute inflammatory states.

Beyond its role in redox control, mGSH directly regulates cell fate in terms of cell death [180]. Under moderate stress conditions with sufficient ATP availability, mGSH depletion favors activation of the intrinsic apoptotic pathway through the release of cytochrome c and other pro-apoptotic proteins, facilitated by cardiolipin oxidation, which weakens its binding to cytochrome c, and by oligomerization of Bcl-2 family proteins such as Bax and Bak. Once in the cytosol, cytochrome c promotes apoptosome formation, activating caspases and triggering controlled apoptosis [181,182]. However, when mitochondrial damage is massive and accompanied by severe energetic collapse, the cell cannot sustain the biochemical processes required for apoptosis, activating alternative cell death mechanisms. One such mechanism is ferroptosis, a regulated form of cell death which, although it requires minimal ATP levels for development, is driven by iron accumulation and uncontrolled lipid peroxidation in an environment of insufficient GSH and glutathione peroxidase 4 (GPx4) dysfunction [183,184,185]. In sepsis, excess iron facilitates the Fenton reaction, increasing ROS production and promoting lipid peroxidation, a key process for ferroptosis activation [186]. Several studies have demonstrated that ferroptosis modulates immune responses in various cell types, both immune and non-immune, contributing to sepsis progression and severity [187]. Wang et al. identified that sepsis-associated encephalopathy induces ferroptosis in the hippocampus, evidenced by increased levels of proteins associated with this pathway, such as GPX4, ACSL4, and SLC7A11 [188]. Decreased GPX4 leads to accumulation of lipid peroxidation products that damage cellular membranes and accelerate sepsis progression [189]. Additionally, the transcription factor p53 regulates some genes related to the cellular response to oxidative stress and ferroptosis, while the Nrf2/HIF-1/TF pathway plays a protective role in LPS-induced sepsis [190].

In contrast, necrosis represents a form of non-programmed, passive, and irreversible cell death that occurs when cellular energy is nearly depleted. Necrosis manifests as abrupt loss of cell membrane integrity and massive release of proinflammatory signals (DAMPs), triggering an exacerbated systemic inflammatory response and severe tissue damage [191]. Therefore, mGSH depletion not only compromises antioxidant control but marks a critical point in mitochondrial dysfunction. By altering redox homeostasis and the OXPHOS system, it impairs basal respiration and reduces ATP production [164,192]. Mitochondria cease to efficiently generate energy and become uncontrolled sources of ROS, perpetuating the inflammatory process, disrupting intracellular redox homeostasis, and propagating cell death signals. The functional consequences of mitochondrial dysfunction on organ physiology have been demonstrated in experimental models of glutathione depletion [193]. These bioenergetic alterations affect various cell lineages and precipitate a scenario of hypoperfusion, lactic acidosis, and multiorgan failure closely linked to the prognosis of this condition.

### 4.3. Glutathione and Organ Failure in Sepsis: Clinical Evidence and Prognostic Implications

Despite significant advances in the understanding of sepsis, the precise mechanisms driving organ dysfunction remain incompletely defined. Microcirculatory alterations have been described [194]; however, both clinical and experimental observations indicate that, despite adequate or even elevated tissue oxygen levels [195], cells exhibit a reduced capacity to utilize oxygen [196]. A reduction in cellular oxygen consumption and minimal evidence of cell death have been reported [197], suggesting that the underlying issue lies in intracellular oxygen utilization rather than its delivery. This highlights the need for a revised paradigm that explains how organ dysfunction may develop in the absence of overt structural damage and despite sufficient oxygen availability [198]. Consequently, multiple organ dysfunction syndrome (MODS) in the context of sepsis should be interpreted less as a consequence of irreversible structural injury and more as an adaptive, functional, and potentially reversible response [199].

Mitochondria, which are responsible for nearly all oxygen consumption used in ATP production, represent the epicenter of this bioenergetic dysfunction. Studies in patients with septic shock analyzing skeletal muscle biopsies have demonstrated a significant reduction in the activity of electron transport chain (ETC) complexes I to IV, particularly complex I, which inversely correlates with vasopressor requirements and plasma nitric oxide levels [32]. Patients with a fatal outcome exhibited lower mitochondrial ATP levels and a marked reduction in mitochondrial glutathione (GSH), indicating a profoundly altered redox state. Since mGSH is essential for ROS neutralization and for preserving ETC function, its depletion promotes mitochondrial dysfunction and bioenergetic failure. Supporting this, Japiassu et al. demonstrated a significant reduction in state 3 mitochondrial respiration (coupled to ATP synthesis) in monocytes from patients with septic shock compared to non-septic surgical controls (5.6 vs. 9.89 nmol O_2_/min/10^7^ cells; *p* < 0.01) [146]. This functional impairment inversely correlated with organ dysfunction severity, as assessed by the SOFA score (r = −0.46; *p* = 0.005), reinforcing the link between mitochondrial bioenergetic failure and the degree of multiorgan dysfunction. Collectively, these findings identify mitochondrial dysfunction and mGSH depletion as key contributors in the pathophysiology of MODS.

However, most of these data originate from animal models or in vitro studies, whose capacity to accurately reflect the human pathophysiological context is limited. One of the main methodological limitations lies in the routine use of isolated cells or mitochondria from septic tissues to assess ETC activity, a process that may neglect the influence of key circulating factors such as cytokines [200]. Supporting this concern, Boulos et al. demonstrated that serum from patients with septic shock induces pronounced mitochondrial dysfunction in isolated healthy mitochondria, highlighting the direct pathogenic role of circulating factors. Furthermore, they found that inhibition of nitric oxide synthesis and poly (ADP-ribose) polymerase (PARP) activity can mitigate this mitochondrial damage [201]. These findings underscore the limitations of current experimental models and emphasize the need for studies in human patients to comprehensively assess the impact of circulating factors on oxidative stress and organ dysfunction during sepsis.

On this regard, the role of glutathione becomes particularly relevant, given its essential function in mitochondrial antioxidant defense and redox regulation during sepsis. Most available data regarding glutathione level changes stem from experimental animal models. In rats, glutathione synthesis rates increased in tissues such as the lung, heart, muscle, liver, and spleen in response to infection, except in blood, where the rate remained unchanged [202]. However, prolonged stress beyond three days led to hepatic glutathione depletion [32]. Clinical data in humans also support its relevance. In a human endotoxemia model where healthy volunteers were administered *Escherichia coli* lipopolysaccharide (LPS), a significant drop (24–32%) in total plasma glutathione concentrations was observed between 3 and 4 h post-administration, without detectable changes in GSH synthesis in muscle or peripheral blood [203].

In agreement with these findings, Andresen et al. analyzed GSH kinetics in patients with septic shock using spectrophotometric measurements in red blood cells [204]. Their results demonstrated a significant decline in GSH levels during the first 24 h and throughout the first week. Concurrently, total glutathione levels in red blood cells were tripled at admission and continued to rise over the first week, reflecting a compensatory attempt by the organism to restore redox balance through increased antioxidant reserves. At three months, both parameters returned to normal, suggesting that restoration of redox equilibrium is associated with favorable clinical recovery.

The association between sepsis severity and the antioxidant response has also been investigated, although most studies focus on total antioxidant capacity (TAC) due to its methodological simplicity and widespread availability. Studies such as that by Tsai et al. report a weak but statistically significant positive correlation between TAC values and multiorgan dysfunction (r = 0.43; *p* < 0.01) [205], while other investigations have demonstrated a direct association between TAC levels and the APACHE II score, in both critically ill and septic patients [10,206].

Nevertheless, few studies in the literature distinguish between glutathione fractions and their specific relationship with organ failure. Although quantification of reduced glutathione is technically challenging, some reports have shown lower levels of GSH and GSSG at diagnosis in patients with worse clinical outcomes, though without statistical significance [207]. Additionally, a progressive decline in GSH and the GSH/GSSG ratio has been observed in non-survivors, while levels remained stable in survivors. From another perspective, a classic study by Ogilvie et al. reported reduced plasma glutathione peroxidase activity in non-surviving patients compared to controls; however, the authors noted that this reduction may not necessarily reflect a functional deficiency at the tissue level [208]. Hsiao et al. observed increased lipid peroxidation (TBARS) and decreased GPx activity in non-surviving septic patients, highlighting antioxidant failure during sepsis [209]. More recently, an observational study in patients with vasodilatory shock found that plasma glutathione peroxidase (GPx) activity at admission was inversely correlated with lactate levels and SOFA scores, reinforcing the hypothesis that the GSH–GPx system is directly involved in regulating organ damage induced by oxidative stress in sepsis [210]. Similarly, Kim et al. reported that decreased plasma and erythrocyte glutathione reductase activity in non-survivors was associated with increased 28-day mortality, highlighting the prognostic significance of glutathione-related enzymes in septic shock [174].

In relation to the impact of antioxidant activity on mortality in patients with sepsis, the two multicenter studies conducted by Lorente et al. represent, to date, the largest cohorts to have assessed redox balance in this clinical context [211,212]. These studies demonstrated significantly higher levels of total antioxidant capacity (TAC) in non-surviving patients, both at ICU admission and during the first week of follow-up, and further showed that this elevation was independently associated with mortality. The authors interpreted these findings as a compensatory antioxidant response to intense oxidative stress in patients with poor clinical outcomes. However, the majority of prior studies have reported significantly lower plasma TAC levels in non-survivors compared to survivors with severe sepsis [8,9,213]. This discrepancy has been attributed, in part, to methodological differences, particularly the use of the Total Radical-Trapping Antioxidant Parameter (TRAP) assay, which measures the ability of plasma antioxidants to neutralize peroxyl radicals generated by 2,2′-azobis (2-amidinopropane) dihydrochloride (ABAP) [214]. This method has a notable bias, as uric acid may account for 47% to 76.4% of the TAC measured, thereby limiting its validity as a comprehensive biomarker of redox status (for a summary, cf. Table 1).

In line with this limitation, Mackinnon et al. observed in a cohort of 50 critically ill patients that both TAC and uric acid levels were significantly elevated in non-survivors, leading to the hypothesis that this increase may result from a secondary elevation in uric acid associated with renal dysfunction [206]. Previously, Tsai et al. reported similar findings in patients with systemic inflammatory response syndrome (SIRS), identifying uric acid, vitamin C, vitamin E, and other antioxidants as the main determinants of TAC variations measured by the TRAP method (*p* < 0.001 for each factor) [205]. These findings have been corroborated by subsequent studies, reinforcing the need for cautious interpretation of TAC values obtained through this technique, particularly in clinical scenarios involving impaired renal function.

**Figure 3:**
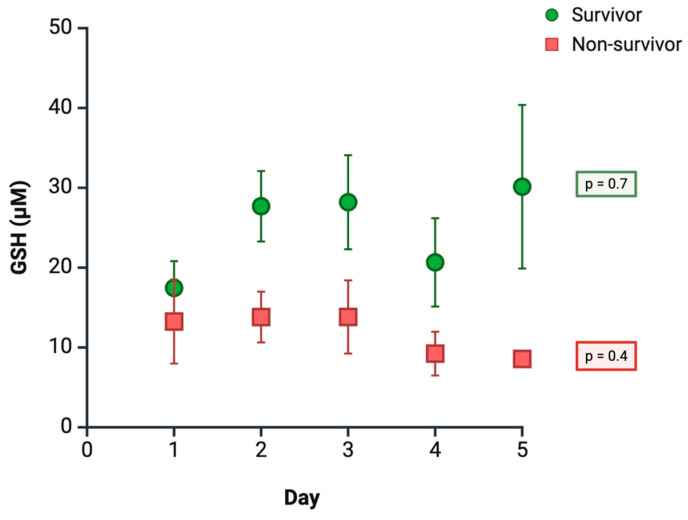
**Time course of plasma GSH levels according to clinical outcome.** Data are expressed as mean ± Standard Error of the Mean (SEM) (survivors: n = 54; non-survivors: n = 18).

In light of the aforementioned methodological limitations and reported inconsistencies, we present preliminary results from our own group, as-yet unpublished study, which evaluated plasma GSH levels using high-performance liquid chromatography (HPLC) in a cohort of 72 septic ICU patients, followed for five days. The most relevant findings indicate that patients who died (n = 18) consistently exhibited lower GSH levels compared to survivors (n = 54), although these differences did not reach statistical significance on individual follow-up days. Throughout the observation period, GSH levels remained relatively stable; however, by day 5, an upward trend was observed in survivors, whereas patients with unfavorable clinical outcomes showed a continued progressive decline (Figure 3). Multivariate analysis of repeated measures confirmed that a decrease in mean GSH levels was independently associated with an increased risk of mortality (OR 0.9; 95% CI 1.2–6.7; *p* = 0.02), after adjustment for age and SOFA score. These findings provide additional evidence supporting the role of GSH in the clinical course of sepsis and reinforce the positive results previously obtained in our study.

Therapeutic efforts in sepsis have traditionally focused on optimizing tissue oxygenation and modulating the dysregulated inflammatory response. However, such interventions have not led to a substantial improvement in survival [1,215]. This therapeutic failure has highlighted that organ dysfunction in sepsis is not solely due to tissue hypoperfusion, but also to impaired cellular oxygen utilization [68]. Consistent with this paradigm, some clinical studies have reported a positive correlation between oxidative stress, mitochondrial dysfunction, and disease severity in septic shock [32,69,70,71]. Based on this understanding, advances in redox biology have identified new therapeutic targets focused on modulating oxidative stress, with antioxidant therapy emerging as one of the most extensively studied strategies. Nonetheless, no study to date has provided conclusive evidence regarding the beneficial effects of antioxidant supplementation in critically ill patients [216,217]. One possible explanation is that conventional antioxidants are distributed nonspecifically throughout the body and fail to reach effective concentrations within mitochondria—the primary site of ROS generation.

In this regard, a 2012 Cochrane review evaluated the efficacy of intravenous N-acetyl-cysteine (NAC) in adult patients with systemic inflammatory response syndrome (SIRS) or sepsis and concluded that no significant differences were found compared to placebo in terms of mortality, ICU length of stay, mechanical ventilation duration, or incidence of new organ failure. Furthermore, delayed administration of NAC was associated with cardiovascular instability, suggesting a potential adverse effect in the later stages of sepsis [218]. The proposal to use mitochondria-targeted antioxidants as a treatment for sepsis holds significant potential. Although preliminary results are promising, further research is needed to confirm their clinical efficacy [219].

### 4.4. Glutathione as a Potential Therapeutic Option in Adult Patients

The different possibilities to increase cellular glutathione in a clinical attempt to better treat or prevent diseases have been recently reviewed [220]. The use of GSH itself, its derivatives, NRf-2 activators, cysteine prodrugs, foods, and special diets all might have advantages and drawbacks that are thoroughly discussed therein. Strategies involving administration of NAC—a GSH precursor and the most widely used antioxidant in both clinical studies and medical practice—have clear limitations, as they increase extracellular or cytosolic GSH levels but do not effectively replenish the mitochondrial pool. At present, no consensus exists regarding the optimal strategy for targeting these compounds, but strengthening mitochondrial antioxidant defenses in the septic context is theoretically considered a valid and promising approach.

## 5. Conclusions and Future Perspectives

Accumulated evidence places glutathione at the center of the pathological mechanisms that govern the clinical evolution of sepsis. Its ability to regulate redox balance, modulate immune responses, preserve endothelial integrity, and maintain mitochondrial function makes it a key player in the transition from adaptive response to the functional collapse characteristic of multiple organ dysfunction syndrome (MODS). GSH depletion during sepsis is not an isolated phenomenon, but rather an integrated manifestation of metabolic, immunologic, and oxidative disturbances converging in cellular failure. Nevertheless, despite its demonstrated biological relevance in experimental models, the clinical translation of glutathione relevance remains limited. Human studies face major methodological constraints and produce heterogeneous results, preventing firm conclusions regarding its utility as a biomarker or therapeutic target. In this context, the preliminary findings from our ICU cohort—suggesting a potential prognostic value of plasma GSH levels measured by HPLC—open a promising avenue for further investigation and validation. Looking forward, there is a need to move toward more specific and personalized therapeutic strategies aimed at effectively restoring intracellular and mitochondrial GSH pools or enhancing endogenous synthesis. This requires not only optimizing delivery routes and compound selection, but also identifying patient subgroups with altered redox profiles who may benefit differentially. The integration of dynamic redox biomarkers with clinical and molecular parameters may represent a paradigm shift in the stratification and treatment of sepsis. Ultimately, glutathione should not be understood merely as an antioxidant, but as a functional integrator linking oxidative stress, cellular metabolism, and immunity. Its study offers a promising path toward redefining diagnostic and therapeutic approaches in sepsis—a condition that continues to impose an unacceptably high burden of mortality and morbidity worldwide.

## Figures and Tables

**Table 1:** **Clinical** **investigations of glutathione levels and antioxidant capacity in septic patients**.

Author	Design and Sample	Objetive	Biomarkers	Sample/Method	Measurement Time	Results
**Ogilvie et al., 1991** [208]	Prospective observational; adults with septic shock (n = 12)	Assess oxidative stress and antioxidants in septic shock	MDA, fluorescent lipid peroxidation products, α-tocopherol, selenium, GPx, conjugated dienes	Plasma; spectrophotometry/HPLC	UCI admission, first 6 h	↑ MDA, ↓ antioxidants; oxidative imbalance associated with worse prognosis
**Goode et al., 1995** [8]	Prospective observational; adults with septic shock (n = 16)	Assess antioxidant status and lipid peroxidation in patients with septic shock and their relationship with organ dysfunction.	Retinol, α-tocoferol, β-caroteno, lycopeno, TBARS, nitrites	Plasma; colorimetry/HPLC	≤24 h frome diagnosis	↓ Antioxidant vitamins, ↑ TBARS and nitrites; correlation between lipid peroxidation and antioxidant deficiency; association with organ dysfunction
**Cowley et al., 1996** [9]	Prospective cohort; adults with severe sepsis and organ dysfunction (n = 15)	Determine plasma antioxidant potential and its relationship with prognosis	Plasma antioxidant potential	Plasma; UV spectrophotometry	≤16 h from onset of organ dysfunction; days 2, 3, 4, 6, 8, 10, 15	↓ Initial antioxidant potential. Normalization or increase to supranormal values in survivors, persistence of low values in non-survivors, correlating with worse prognosis
**Fläring et al., 2005** [203]	Prospective descriptive study; adults with multiple organ failure and ICU stay ≥ 6 days (n = 11). Reference groups: n = 21 COPD, n = 10 healthy controls	Evaluate temporal changes in total and reduced glutathione	Total and reduced glutathione	Whole blood and plasma; HPLC	Every 72 h for 6–15 days	↓ Glutathione in whole blood; ↑ plasma glutathione in patients with multiple organ failure compared with reference groups
**Chuang et al., 2006** [10]	Prospective observational study; severe sepsis (n = 73) and controls (n = 76)	Relate TAC to severity (APACHE II)	TAC; uric acid; bilirubin; albumin	Serum; TRAP	Day 1 of sepsis diagnosis	↑ TAC in severe sepsis; correlated with APACHE II and mortality
**Huet et al., 2008** [70]	Prospective study; adults with septic shock (n = 15). Healthy controls (n = 10)	Evaluate endothelial oxidative stress and GSH depletion	GSH, ROS, RNS, catalase and SOD activity, cell death	Plasma and human umbilical vein endothelial cells (HUVEC); spectrofluorimetry, YOPRO staining, and MTT assay	First 24 h of septic shock	↓ Intracellular GSH in HUVEC; ↑ ROS and cell death; ROS and cell death reduced with N-acetylcysteine or GSH pretreatment; no changes in RNS
**Andresen et al., 2008** [204]	Prospective study; adults with septic shock (n = 21)	Evaluate oxidative damage and its relationship with disease severity	BARS, protein carbonyls, methionine sulfoxide, FRAP, TRAP, vitamin C, vitamin E, bilirubin, uric acid, red blood cell glutathione	Plasma and red blood cells; spectrophotometry, HPLC	ICU admission, 24 h, 72 h, day 7, and 3 months	↑ TBARS and red blood cell glutathione; ↓ vitamin C and reduced glutathione; ↑ bilirubin and uric acid; correlation between ↑ TBARS and sepsis severity
**Karapetsa et al., 2013** [207]	Prospective pilot study; adults with septic shock (n = 17)	Evaluate variability of oxidative stress during sepsis progression	TBARS, TAC, protein carbonyls, reduced and oxidized glutathione, catalase activity	Erythrocytes/plasma; HPLC/spectrophotometry	Days 1, 3, 5, and 8 after sepsis onset	↑ TBARS and protein carbonyls, ↓ reduced GSH, ↓ TAC, and ↓ catalase activity in non-survivors
**Lorente et al., 2015** [211]	Prospective multicenter study; adults with severe sepsis (n = 213)	Relationship between TAC and 30-day mortality in severe sepsis	TAC, MDA	Serum; TRAP and TBARS methods	Day 1 of ICU admission	↑ TAC and MDA in non-survivors; TAC levels associated with higher 30-day mortality
**Kim et al., 2016** [174]	Prospective study; adults with septic shock (n = 60)	Evaluate the relationship between plasma GR activity and mortality in septic shock	Glutathione reductase (GR) activity	Plasma and red blood cells; spectrophotometry	0 h and 24 h post-admission	↓ GR in non-survivors; 24 h decrease associated with ↑ 28-day mortality; positive correlations between plasma and erythrocyte GR, inverse correlation with GSH/GSSG
**Lorente et al., 2018** [212]	Prospective multicenter study; adults with severe sepsis (n = 319)	Evaluate the relationship between TAC during the first week of sepsis and 30-day mortality	TAC and MDA	Serum; TRAP and TBARS methods	Days 1, 4, and 8 of ICU admission	TAC during the first week associated with lipid peroxidation, sepsis severity, and 30-day mortality
**Hsiao et al., 2020** [209]	Prospective study; adults with sepsis (n = 100)	Evaluate the evolution of oxidative stress and antioxidants with clinical outcomes	TBARS; total GSH; GPx activity	Whole blood/erythrocytes; TBARS by spectrophotometry, GSH and GPx by HPLC	Days 1, 4, and 7	↑ Oxidative stress and ↓ GPx in non-survivors; TBARS predicts mortality and is associated with hospital length of stay
**Semedi et al., 2023** [210]	Prospective single-center study; adults with vasodilatory shock (including sepsis) (n = 34)	Evaluate whether GPx activity is associated with shock severity and clinical outcomes	GPx activity	Serum; ELISA	At admission and 24 h	GPx activity does not predict mortality, but is inversely associated with lactate and SOFA score, reflecting vasodilatory shock severity

**Abbreviations**: APACHE II, Acute Physiology and Chronic Health Evaluation II; COPD, chronic obstructive pulmonary disease; ELISA, enzyme-linked immunosorbent assay; FRAP, ferric reducing ability of plasma; GSH, reduced glutathione; GSSG, oxidized glutathione; GPx, glutathione peroxidase; GR, glutathione reductase; HPLC, high-performance liquid chromatography; HUVECs, human umbilical vein endothelial cells; ICU, intensive care unit; MDA, malondialdehyde; MTT, 3-(4,5-dimethylthiazol-2-yl)-2,5-diphenyltetrazolium bromide; ROS, reactive oxygen species; RNS, reactive nitrogen species; SOFA, sequential organ failure assessment; TBARSs, thiobarbituric acid reactive substances; TAC, total antioxidant capacity; TRAP, total radical-trapping antioxidant parameter; UV, ultraviolet; YOPRO, fluorescent dye for apoptosis detection.
